# Encysted Calculus

**Published:** 1853-08

**Authors:** 


*Encysted Calculus, Severe Symptoms of Stone in the Bladder fo*[illegible] *Several Years; Imperfect Evidence of the presence oj the Calculus. Death. Cyst of the Bladder containing the Foreign Body.*—There is a rule respecting the operation of lithotomy to which operators genarally adhere—viz., never to cut for stone unless the presence of the calculus shall have been distinctly made out by percussion with the staff a few moments before the operation. This is an excellent rule, and it may be surmised that more than once both surgeon and patient have been saved the anno[illegible]ance of a useless operation.

The case which we have this day to bring before our readers offers a beantiful illustration of the soundness of the above-mentioned practical caution handed down to us by our forefathers; for the post-mortem examination showed very plainly that, althrough there was really a stone in the bladder, none of the means at our command could have accomplished its extraction, even with a very large incision.

We happen to have witnessed two operations of lithotomy in charitable institutions of this metropolis, where no stone could be found; and it must be confessed, that the rule just alluded to was not, in in these instances, strictly followed. But it is, on the other hand, quite certain that the evidence by percussion is sometimes extremely faint; and it may happen that a surgeon will hear the sound, whilst his colleagues cannot perceive it. The course to be pursued in such a case is not easly settled, but we may cite, as an encouragement for operating, a case sometime ago under the care of Mr. Le Gros Clark, at St. Thomas's Hospital. The little boy (about six years of age) had on a previous occasion been removed from the theatre without being operated upon, because no stone could be heard. When the patient was put upon the table for the second time, some of the surgeons present heard the stone, and others not. Mr. Clark was among the former; Lithotomy was performed, and Mr. Clark had the satisfaction of removing, by means of the scoop, a stone of the size of a pea.

We recollect an operation of lithotomy, performed a few months ago at Guy's Hospital, by Mr. Hilton, in which it appeared, when the forceps had been used for some little time, that the bladder contained no stone, although percussion had yielded the usual sound. But by dint of perseverance and well-directed efforts, Mr. Hilton succeeded in dislodging a moderately large calculus from above the pubes, in wh ch locality it was firmly held by the muscular fibres of the bladder, or a small cyst.

Mr. Cock, at the same hospital had, on another occasion, a great deal of trouble in extracting a mulberry calculus, which was firmly grasped by the vesical mucous membrane and muscular coat, the anfractuosities of the stone being larger and more prominent than usual.

In Paris, M. Roux perfomed, some years ago, the lateral operation, in a case where the stone was distinctly heard; but no calculus could be found, and it was supposed that the foreign body was encysted somewhere behind the pubes. At all events some advantage might be derived from using the stethoscope more frequently when there is any donbt respecting the diagnosis, as advised by Skoda in the work lately translated by Dr. Markham. Still it will be seen by the following case, (for the details of which we are indebted to Mr Gamgee, house-surgeon to the hospital,) that even the sound yielded by the instrument striking against the calculus may deceive, and that the stone may be grasped in such a manner as to preclude the possibility of extraction.

John W—, aged forty-eight, was admitted Jan. 10, 1850, under the care of Mr. Arnott. The patient had left the army two years previously, was married, and of regular habits. No malignant disease can be traced in the family. The patient suffered from inflammation of the testicle about twenty-two years before the date of this admission, and was six or seven years afterwards tapped for hydrocele; he also had gonorrhœa many years back, for which he used no injections. For two years and a half preceding his application at the hospital, the patient had very severe symptoms of irritated bladder, with occasional passage of blood; and on admission, the symptoms were *bonâ fide* those of vesical calculus. A week before the man was admitted, he passed, without pain, a small stone, about the size of a pea. A few days before he came into the house, five ounces of water were injected into the bladder; No. 9 sound was introduced; and on pushing it well into the viscus, the convex portion seemed to touch something rough; and on turning the point suddenly in the direction of the supposed foreign body, a distinct chink was produced. The urine was fœtid, turbid, pale, and slightly acid, and threw down a mucous deposit after standing. Under the microscope, phosphatic crystals, some pus, ann mucous corpuscles, were seen.

Mr. Arnott ordered one ounce and a half of infusion of pareira to be taken three times a day, and ten grains of Dover's powder at night. The symptoms for the next fourteen days became rather aggravated, notwithstanding injections of warm water and decoction of poppies into the bladder, and in spite of pretty large doses of opium, hyoscyamus, and nitric acid. The patient used to pass urine about fifteen times in the night.

The symptoms of stone became more and more urgent; the bladder was therefore examined by Mr. Arnott, Mr. Quinn, and Mr. Erichsen. The sound could not be turned to the right side so freely as to the left, this circumstance giving the idea of the existence of some growth on that side; greater pain was also experienced when the part was touched, but no stone could be felt, though the bladder was carefully explored. On pushing the sound well in, and turning it to the right side, the instrument did not seem to go beyond the projection just mentioned, and seemed to reach the point where a chink was before obtained. All medicines were left off, except opium enemata.

Twelve days after this, (five weeks from the admission,) the man, whose symptoms varied but little, was sounded again; the difficulty of turning the instrument to the right side seemed to be rather increased, but some allowance was made in consequence of there being very little water in the bladder; the instrument, when left to itself, had a direction to the left side.

Six days afterwards, the patient was sounded for the third time, with three ounces of water in the bladder; great suffering was experienced, but no stone felt.

Four days subsequently to this examination, chloroform was given, and the patient taken into the theatre. Three ounces of fluid were thrown into the bladder; but as no stone could be felt, the projected operation was not performed.

Up to the 22nd of March, (ten weeks after admission,) the bladder remained irritable, and the urine alkaline, though the patient was well supported with brandy, and took benzoic acid.

At this period small doses of copaiba were ordered; the urine thereupon became less alkaline, and the mucous deposit diminished considerably.

On the 30th of March, a week after this change of medicines, the patient only rose four times in the night, whilst a short time previously he used to be disturbed more than thirty times from his sleep.

Fifteen weeks after admission, April 18, 1850, he was so far improved that he wished to be discharged. The man presented himself several times at this hospital between the latter date and his finally entering the house in November, 1852. Mr. Erichsen sounded him repeatedly without success, except on one occassion, in the earlier part of the summer of 1851, when the stone was distinctly struck by the instrument.

The great frequency and pain of micturition caused great debility; and about one month before death, pleurisy was diagnosed on the left side and treated by blisters. Four days before the patient's death, a tumour nearly as large as the first showed itself on the left side of the thorax towards the lower part. It became a question whether this distinctly fluctuating tumour was due to escape of fluid from the chest. A puncture was made into it, and exit given to rather more than half a pint of very fœtid, brownish pus.

After this period the patient gradually sunk, and died Dec. 6, 1852. It is important to notice that the man was treated a few months before his second and last admission for a slight attack of jaundice.

The post-mortem examination was conducted by Mr. Gamgee, and took place thirty-three hours after death. On opening the thorax, the left pleura was found to contain 136 ounces of turbid serosity, with a few flocculi floating in it; the right pleura contained about a pint and a half of the same fluid. The lungs were studded at the apices by tubercles of the size of small peas, and the organs adherent to the parietal pleuræ. An incision being accidentally made into the upper portion of the pericardium, thin pus exuded from that bag. The latter was now cut carefully open anteriorly, and was found to contain sixteen ounces of purulent matter. Pus was seen exuding through the lower portion of the pericardium, and also through the diaphragm, about three inches behind the costal margin. The aperture allowing of this escape was of a circular shape, and capable of admitting the end of a goose-quill; the margin measured about a line in thickness, and was of a deep red colour. The whole surface of the heart was roughened by deposits of lymph, but the internal aspect of the organ was normal. No pathological change was noticed in the kidneys, save a very marked congestion. On the right lobe of the liver were seen several inequalities, resulting from contractions after deposition of lymph. The external margin of the left lobe was completely adherent to the false ribs, and in this locality a large cavity was observed, capable of admitting a good-sized fœtal head. This cyst contained pus, and communicated by an aperture, about the size of a goose-quill, with the pericardium, and also with the integuments by an oblique opening at the edge of the false ribs, where the abscess had pointed, and had been opened.

The bladder, upon which organ much interest was centred, was carefully dissected, and presented the following appearances:—Prostate gland healthy; bladder proportionally small; at the upper part, and on the right side, is a tumour projecting directly outwards, about the size of a pullet's egg. In a groove on the posterior portion of this protuberance, the right ureter is seen coursing. On manipulating this tumour, it is felt to contain calculous matter; and on cutting into the swelling from without inwards, two whitish, slightly roughened stones are found. In this locality, the coats of the bladder are at least three times the thickness of the other parts of the organ. The latter was now opened by a longitudinal incision; and on examining the mucous surface, a small aperture, capable of containing the extremity of a middle-sized catheter, was obseved. This opening was situated about half an inch above the point of entrance of the right ureter, was perfectly circular in shape, and led into the cavity above described, which contained two calculi. On carefully examining this tumour, it seemed manifest that it was formed by a kind of cyst attached to, and communicating with, the bladder, which cyst had perhaps gradually taken its development around the calculi, and grown outwards, becoming subsequently considerably thickened.

It should now be observed, that the aperture within the bladder was just large enough to admit the extremity of the staff, and hence arose the distinct sound which had repeatedly been heard on the introduction of the instrument. When no chink could be produced, on the day when the patient was sounded in the theatre, as also on other occassions when no evidence of stone could be made out, it is very probable that the instrument either did not exactly come in contact with the small aperture communicating with the cyst, or that the opening was momentarily covered with mucus. It is evident that the patient might have been cut for stone, and no calculus been found, if, on the day he was brought to the theatre, the instrument had just struck on the quarter of an inch square of calculus surface which the cyst left uncovered; so that the rule which we mentioned in our introductory remarks would, in this instance, have failed to prevent a useless operation.

Besides the view which we just took of the formation of the cyst, it might be supposed that the latter existed originally or congenitally in the bladder, and that the calculi were the consequence of the stagnation of the urine in the pouch. By the post-mortem examination, it is now also clear why irritable bladder was the principal symptom presented by the patient. No foreign body was rolling in the vesical cavity, no stone stopped up the urethra suddenly, and therefore the main cause of distress was the presence of the calculus, seemingly in the coats of the bladder, constantly irritating and dragging the organ. Nor did the stagnation of urine in the cyst, and its ever recurring decomposition, as well as the imperfect action of the coats of the bladder, contribute in a small degree to the patient's distress.

The pathological changes discovered in the bladder, taken in connexion with the symptoms and the difficult diagnosis, are of so interesting a nature, and have taken up so much of our space that we hardly have room to allude to the hepatic abscess which, in this patient, opened into the pericardium. The course here taken by the purulent matter is of a rather unusual kind; and it is somewhat strange, considering the quantity of pus which was found in the pericardium, that death did not occur more suddenly, the more so as one of the pleural sacs contained a large amount of serum. There may have been som connexion between the attack of jaundice and the hepatic abscess which subsequently formed, but the data are not sufficiently accurateto permit of any speculation on the subject.—*London Lancet.*

